# Disassociation of Muscle Insulin Signaling and Insulin-Stimulated Glucose Uptake during Endotoxemia

**DOI:** 10.1371/journal.pone.0030160

**Published:** 2012-01-20

**Authors:** Kimberly X. Mulligan, R. Tyler Morris, Yolanda F. Otero, David H. Wasserman, Owen P. McGuinness

**Affiliations:** Department of Molecular Physiology and Biophysics, Vanderbilt University School of Medicine Nashville, Tennessee, United States of America; Paris Institute of Technology for Life, Food and Environmental Sciences, France

## Abstract

Lipopolysaccharide (LPS) elicits a strong immune response, which leads to the release of inflammatory cytokines. Increased cytokine production has been shown to impair insulin-mediated glucose disposal. LPS can alter other factors, such as muscle blood flow and insulin signaling in the myocyte,that can influence glucose disposal. We hypothesize that LPS induced impairments in cardiovascular function contribute to the associated impairments in insulin action *in vivo*. Male wild-type C57BL/6J mice had a catheter implanted in the jugular vein for infusions and the carotid artery for sampling 5 days prior to the hyperinsulinemic-euglycemic clamp. Mice were treated with vehicle, low-(1 ug/gBW) or high-dose (10 ug/gBW) LPS 4 hours prior to the clamp. Muscle glucose uptake (MGU) was assessed using [2-^14^C] deoxyglucose. While both low- and high-dose LPS inhibited insulin-stimulated MGU compared to vehicle-treated mice, the impairment was more significant with the high-dose treatment (∼25% in soleus and ∼70% in both gastrocnemius and vastus lateralis). Interestingly, insulin signaling through the PI3-kinase pathway in the muscle was not affected by this treatment suggesting that the decrease in MGU is not directly due to impairments in muscle insulin action. Echocardiography demonstrated that high-dose LPS treatment significantly decreased stroke volume (∼30%), heart rate (∼35%), and cardiac output (∼50%). These observations were not seen with vehicle or low-dose LPS treatment. High-dose LPS treatment also significantly decreased muscle blood flow (∼70%) and whole body oxygen consumption (∼50%). Thus, *in vivo* acute endotoxemia does not impair insulin signaling through the PI3-kinase pathway in skeletal muscle and decreased tissue blood flow likely plays a central role in the impairment of glucose uptake in the muscle.

## Introduction

Sepsis has been defined as “the systemic inflammatory response to infection” [1]. It is the second leading cause of death in non-coronary intensive-care unit (ICU) patients and a major cause of morbidity and mortality. During sepsis, lipopolysaccharide (LPS), which is derived from the cell wall of gram-negative bacteria, can induce the expression of inflammatory mediators and cytokines such as TNF-α, interleukin-6 (IL-6), interleukin-1β (IL-1β), and interleukin-10 (IL-10), as well as lead to the release of nitric oxide (NO). Common side effects in response to sepsis are hyperglycemia and severe insulin resistance. This is in part due to the role that the immune response plays in the regulation of skeletal muscle glucose uptake (MGU). Skeletal muscle comprises the bulk of insulin-sensitive tissue in the body and is an important site for glucose disposal. Insulin augments MGU by not only regulating insulin-stimulated GLUT4 translocation and subsequent glucose phosphorylation in the muscle but by also enhancing tissue blood flow [2], [3], [4], [5].

Mediators of the inflammatory response have been shown to impair muscle insulin signaling and blood flow, however little work has been done to examine how these factors interact with one another *in vivo*. Youd *et al.* has shown that *in vivo* a 3 hour infusion of TNF-α into anesthetized rats inhibited insulin-stimulated microvascular recruitment and lead to decreased insulin-stimulated hind-leg glucose uptake. However, insulin signaling was not measured in this study [6]. While TNF-α has been shown to impair the vasodilator effects of insulin in skeletal muscle resistance arteries through impairments of insulin activation of AKT in rats [7] and impair insulin signaling and insulin-stimulated glucose uptake in muscle [8], this work was done either *ex vivo* or in cultured cells. *In vivo* Fan *et al.* found that LPS lead to whole body insulin resistance in rats and impaired insulin signaling [9]. Yet a recent *in vivo* study utilizing a hyperinsulinemic-euglycemic clamp in mice was unable to detect an impairment in insulin action following an i.p. LPS injection [10]. Therefore it is unclear how much of the impairments seen with acute inflammatory stress are signaling- and/or cardiovascular–mediated. Interestingly the majority of the work has been done in rats, with limited and apparently different responses in mice.

While any number of factors can contribute to inhibition of insulin-stimulated glucose uptake in the presence of an LPS challenge, there has been little work to examine the relationship between the LPS induced inflammatory response and cardiac dysfunction, insulin signaling, and decreased muscle glucose delivery *in vivo*. There are vascular as well as metabolic events occurring *in vivo* that can affect glucose metabolism. The aim of the current study was to examine the relationship between cardiovascular dysfunction, insulin signaling, and decreased muscle glucose delivery when mice are challenged with LPS *in vivo* utilizing several modifications to more specifically examine the physiological effects on skeletal muscle. We hypothesized that if LPS is accompanied by alterations in the cardiovascular system, alterations in tissue blood flow as opposed to insulin signaling are major contributors to the impaired muscle glucose uptake.

## Methods

### Animal Care and Husbandry

All procedures were performed on male wild-type (WT) C57BL/6J background (Jackson Laboratories, Bar Harbor, ME). At three weeks of age, mice were separated by gender and maintained in micro-isolator cages on a 12-h light/dark cycle with free access to food and water. All experiments were performed in mice at ∼3 months of age. The Vanderbilt University Institutional Animal Care and Use Committee approved all procedures performed.

### Surgical Procedures

The surgical procedures utilized to implant chronic catheters have been described previously [11], [12]. Briefly, mice were anesthetized with isoflurane. The left common carotid artery and right jugular vein were catheterized for sampling and infusing, respectively. The free ends of the catheters were tunneled under the skin to the back of the neck where the loose ends of the catheters were attached via stainless steel connectors to tubing made of Micro-Renathane (0.033 in OD). The tubing was exteriorized and sealed with stainless steel plugs. Animals were individually housed after surgery and body weight was recorded daily.

### In Vivo Metabolic Experiments

**Figure 1:**
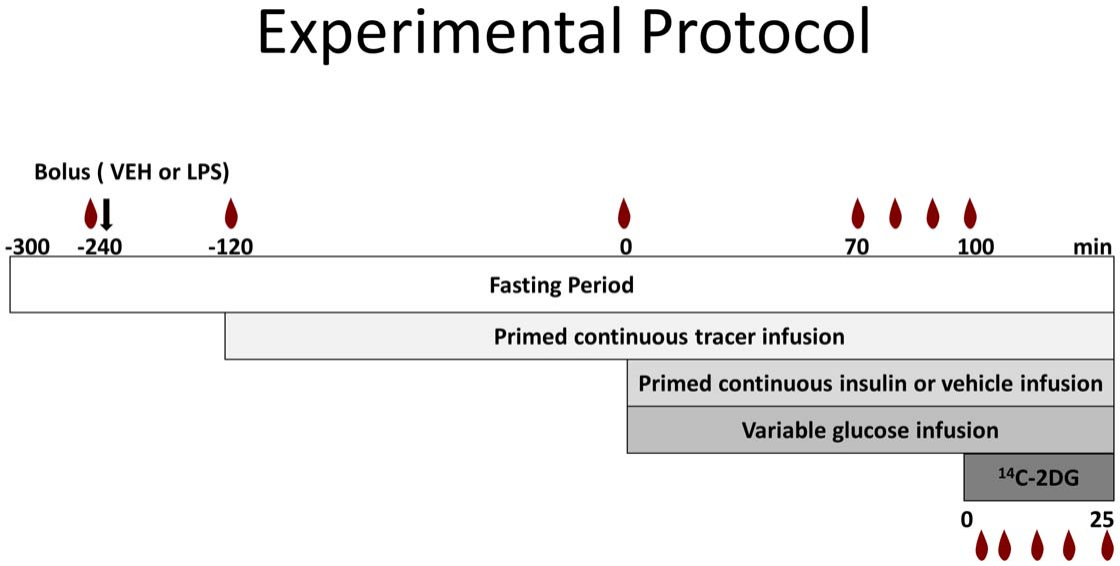
Protocol used during the hyperinsulinemic-euglycemic clamp. On the day of the *in vivo* metabolic experiments, chronically catheterized conscious mice were placed on a 5 h fast (t = −300 min). A bolus of VEH, low- or high-dose LPS was injected at t = −240 min. At t = −120 a primed continuous tracer infusion was begun. At t = 0 an infusion of insulin and glucose or vehicle (i.e. saline) was initiated. After 100 min, 2-deoxy glucose was injected and mice were sacrificed after 25 min.

All metabolic experiments were performed following a ∼5-day postoperative recovery period. Mice were not used in experiments unless their body weight was within 10% of their body weight prior to catheter implantation. For metabolic studies, conscious, unrestrained mice were placed in an ∼1-L plastic container lined with bedding and fasted at 7:00 am (t = −300 min). The mice were immediately connected to a Dual Channel Stainless Steel Swivel (Instech Laboratories, Plymouth Meeting, PA) to allow simultaneous jugular vein infusion and sampling of arterial blood. Mice were not handled and were allowed to move freely to eliminate stress. Prior to vehicle or endotoxin injection (t = −240 min) a blood sample (60 µl) was drawn for measurement of blood glucose and plasma cytokine levels. Mice then received a bolus of vehicle or *E. coli* endotoxin (LPS; 0111:B4) (Lot#129K4025, Sigma-Aldrich, St. Louis, MO) at a dose of either 1 ug/gBW (low-dose) or 10 ug/gBW (high-dose) into the jugular vein catheter. Five µCi bolus of [3-^3^H]-D-glucose was given into the jugular vein 2 h after the mice received the bolus (t = −120 min) followed by a constant infusion at a rate of 0.05 µCi/min. Following a 2 h equilibrium period at t = 0 min (i.e. 4 h after the bolus and a 5 h fast) a baseline arterial blood sample (200 µl) was drawn for measurement of blood glucose, [3-^3^H]-D-glucose, hematocrit, plasma insulin, and plasma cytokine levels. Red blood cells from a donor mouse on a C57Bl/6J background were washed with and reconstituted in an equal volume of 0.9% heparinized saline (hematocrit ∼50%) and infused at a rate of 4 µl/min for the duration of the study to minimize falls in the hematocrit (Figure 1).

A hyperinsulinemic-euglycemic (120 mg/dl) clamp was used to assess insulin action in three groups: vehicle+insulin (4.0 mU/kg/min, n = 8); low-dose LPS+insulin (2.0 mU/kg/min, n = 8); or high-dose LPS+insulin (2.5 mU/kg/min, n = 10). A lower dose of insulin was used in the LPS groups to attempt to match arterial insulin concentrations as initial studies indicated LPS impaired insulin clearance. Arterial blood samples (10 µl) were taken every ten minutes to determine blood glucose levels. At t = 70, 80, 90 and 100 min, blood samples (50 µl) were taken to determine [3-^3^H]-D-glucose. At t = 100 min, a 13 µCi bolus of 2-deoxy [^14^C] glucose ([2-^14^C]DG) was administered into the jugular vein catheter. At t = 102, 105, 110, 115, and 125 min arterial blood (20 µl) was sampled to determine blood glucose, plasma [3-^3^H]-D-glucose and [2-^14^C]DG. At t = 125 min (∼6 h after the vehicle or LPS bolus), a final arterial blood sample (200 µl) was taken to assess circulating hormones and plasma [2-^14^C]DG. The mice were then anesthetized. The soleus, gastrocnemius, superficial vastus lateralis (SVL), liver, heart, and brain were excised, immediately frozen in liquid nitrogen, and stored at −70°C until future tissue analysis. Basal plasma glucose levels and tissue glucose uptake was assessed in three additional groups: vehicle+saline infusion (n = 9), low-dose LPS+saline infusion (n = 6), and high-dose LPS+saline infusion (n = 8). Comparison of these studies to the insulin clamp experiments permits insulin-independent effects of LPS to be defined.

In a separate group of studies the effects of LPS on the acute activation of insulin signaling was examined. Mice were placed on a 5 h fast (t = −300 min) and received either an I.V. bolus of vehicle or high-dose LPS 1 h after the fast began (t = −240 min). Four h later (t = 0 min), mice received an I.V. bolus of insulin (10 U/kg body weight). At t = 10 min, the mice were anesthetized. The gastrocnemius muscle and liver were excised, immediately frozen in liquid nitrogen, and stored at −70°C until future tissue analysis.

### Plasma and muscle sample analysis

Immunoreactive insulin was assayed using a Linco Rat Radioimmunoassay kit (Linco Research, Inc., St. Charles, MO). Cytokines were analyzed using multiplexing Luminex xMAP technology (Luminex, Austin, TX). Plasma samples were prepared using mouse cytokine/chemokine Lincoplex kit (Linco Research, St. Charles, MO). To measure [3-^3^H]-D-glucose plasma samples were deproteinized with barium hydroxide (Ba(OH)_2_) and zinc sulfate (ZnSO_4_), dried, and radioactivity was determined using liquid scintillation counting. Excised soleus, gastrocnemius, SVL, heart, and brain were deproteinized with perchloric acid and then neutralized to a pH of ∼7.5. A portion of the sample was counted ([2-^14^C]DG and [2-^14^C]DG-G-phosphate ([2-^14^C]DGP) and a portion was treated with Ba(OH)_2_ and ZnSO_4_ and the supernatant was counted ([2-^14^C]DG). Both [2-^14^C]DG and [2-^14^C]DG-phosphate ([2-^14^C]DGP) radioactivity levels were determined using liquid scintillation counting.

### Calculations

Glucose flux rates were assessed using non-steady state equations assuming a volume of distribution (130 ml/kg). Tissue-specific clearance (K_g_) of [2-^14^C]DG and an index of glucose uptake (R_g_) was calculated as previously described [13]:
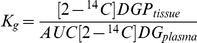


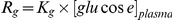
where [2-^14^C]DGP_tissue_ is the [2-^14^C]DGP radioactivity (dpm/g) in the tissue, AUC [2-^14^C]DG_plasma_ is the area under the plasma [2-^14^C]DG disappearance curve (dpm/mL/min), and [glucose]_plasma_ is the average blood glucose (µg/µl) during the experimental period (t = 102–125 min). Data are presented as mean ± SEM and the significance level was set at p<0.05.

### Blood Pressure

Mean arterial blood pressure (MAP) was measured in conscious mice utilizing a Micro-Med, Inc. Blood Pressure Analyzer (Louisville, KY). Baseline blood pressure and heart rate measurements were taken from the carotid artery prior to the LPS injection (t = −240 min) and measurements were repeated 4 h post-LPS injection (t = 0 min).

### Blood Flow

A separate group of mice that were treated with vehicle, low- or high-dose LPS received an infusion of para-amino hippuric acid (PAH). PAH clearance was used to estimate renal blood flow. At t = −240 min animals received a bolus of either vehicle, low- or high-dose LPS. At t = −120 min, animals received a constant infusion of PAH dissolved in saline into the jugular vein at a rate of 24 µg/g/min. Plasma samples were taken at t = −120, 0 (4 h following a bolus of VEH or LPS), 60, and 120 min. The plasma was diluted 1∶5 in Ba(OH)_2_ and ZnSO_4_, centrifuged at 13,000 rpm for 5 min, and the supernatant was diluted 1∶6 in ddH_2_O. The samples were then diluted 1∶6 in the PAH reagent (5 g dimethylaminobenzaaldehyde, 300 mL 95% ethanol, 20 mL 2N HCL, and ddH_2_O to a final volume of 500 mL) and the absorbance of PAH was determined at 465 nm. The PAH clearance (mL/kg/min) was calculated as follows:
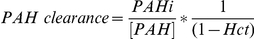

*Where:*

*PAHi = PAH infusion rate (mg·kg^−1^·min^−1^)*

*[PAH] = arterial plasma PAH concentration (mg/ml)*

*Hct = hematocrit ratio*

### Microsphere Isolation

Microspheres were utilized to measure the effect of inflammation on tissue blood flow under basal conditions. Mice were fasted at t = −300 min (∼7:30 am) and at t = −240 min they received a bolus of either vehicle or high-dose LPS. At t = 120 min (6 h following bolus), mice were injected with 50 µL of microspheres (Dye-Trak VII, Triton Technology, INC, San Diego, CA) followed by 50 µL saline into the carotid artery. Mice were anesthetized and the tissues were excised. The tissues were digested overnight in 1 M KOH at 60°C. Following vortex and centrifugation, the pellet containing the microspheres was re-suspended with TritonX-100 and centrifuged. The pellet was re-suspended in ethanol containing 0.2% (v/v) HCL, centrifuged and re-suspended in ethanol. The resulting product was centrifuged and a small amount of ethanol was left on the pellet and allowed to evaporate at room temperature overnight. Cellosolve acetate (250 µL) was added to the microsphere∶ethanol solution to elute the fluorescent dye from the microspheres. The absorbance was determined at 440 and 672 nm. Tissue blood flow was calculated as the product of the ratio of microspheres in a tissue relative to microspheres in both kidneys and PAH clearance (ml/min) divided by tissue weight (ml·g tissue^−1^·min^−1^).

### Echocardiography

Transthoracic echocardiography was performed using a system (Sonos 5500, Agilent, Andover, MA) with a 15-MHz high frequency linear transducer at a frame rate of 100 frames/sec. All images were acquired at a depth setting of 20 mm. Images were acquired at two time points: t = 0 min, which represents the time immediately prior to an injection of LPS and at t = 360 min (6 h following the bolus). The mouse was picked up at the nape of its neck and an ultrasound-coupling gel was applied to the precordium, with the ultrasound probe. Two-dimensional targeted M-mode echocardiographic images were obtained at the level of the papillary muscles from the parasternal short-axis vies and recorded at a speed of 150 cm/s (maximal temporal resolution) for measurements of heart rate. All other measurements were made on screen using the digitally recorded signals. These measurements were used to calculate stroke volume and cardiac output [14], [15]:
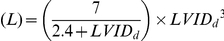


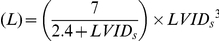


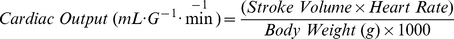
Where LVIDd and LVIDs are left ventricular internal dimensions during diastole and systole, respectively.

### Indirect Calorimetry

Oxygen consumption (VO_2_) was measured by an Oxymax indirect calorimeter (Columbus Instruments, Columbus, OH). Air flow was set at 0.60 l/min and mice were housed individually for 1 day before entering the calorimeter. After a 1 h adaptation to the metabolic chamber, VO_2_ was measured in individual mice for 1 min at 15-min intervals for a total of 48 h under a consistent environmental temperature (22°C). Following 48 h of baseline measurements, food was removed and intraperitoneal injections of vehicle (n = 5), low-(n = 6) or high-dose (n = 9) LPS were administered. VO_2_ was calculated for 8 h following the IP injections. VO_2_ is expressed as the volume of O_2_ consumed per kilogram body weight per hour.

### Western Blots

Protein from skeletal muscle and liver was extracted with a buffer containing 50 mM Tris, 1 mM EDTA, 1 mM EGTA, 10% glycerol, 1% Triton X-100 at pH 7.5. Before using 1 mM DTT, 1 mM PMSF (dissolved in ethanol), 5 ug/mL protease inhibitor cocktail, 10 ug/mL trypsin inhibitor, 50 mM NaF, and 5 mM NaPP were added to the buffer. The tissues were homogenized on ice and centrifuged for 20 min in the cold room. The protein extract was collected and stored at −80°C until used. Protein extracts (20 µg) were combined with NuPage LDS sample loading buffer (4X), NuPage reducing agent (10X) (Invitrogen, Carlsbad, CA), and distilled water up to 25 µL. This was placed in a 75°C water bath for 10 min, and size-fractionated by electrophoresis on 10% SDS-polyacrylamide gel (Invitrogen, Carlsbad, CA). Proteins were transferred from the gel to a PVDF transfer membrane by electroblotting. Membranes were incubated at room temperature with 5% nonfat dried milk, in Tris-buffered saline-Tween (TBS-T) (Sigma, St. Louis, MO) for 2 h, washed 3X for 5–10 min in TBS-T, and then incubated overnight at 4°C with mouse anti-pAkt (Ser^473^) antibody (1∶1,000), mouse anti-Akt antibody (1∶500), mouse anti-pGSK (Ser^9^), mouse anti-GSK antibody (1∶1,000) mouse anti-IRS-1 (1∶1000), mouse anti-pIRS-1 (Tyr^895^), or mouse anti-pIRS-1(Ser^307^) (all antibodies purchased at Cell Signaling Technology, Danvers, MA). After incubation, samples were washed 3X for 5–10 min in TBS-T, incubated with peroxidase-conjugated secondary antibody, and analyzed using enhanced chemiluminescence (GE Healthcare, Piscataway, NJ).

### Real Time PCR

Total mRNA was extracted from the skeletal muscle and liver using the RNeasy fibrous tissue kit (Qiagen Sciences, Germantown, MD). cDNA was synthesized from 2.5 µg RNA with the SuperScript III first-strand synthesis system first-strand cDNA synthesis kit (Invitrogen, Carlsbad, CA). PCR amplification reactions were performed in triplicate using the ABI detection system (Applied Biosystems, Foster City, CA) and the ΔΔCT method was used to quantify mRNA levels. Gene expression was normalized to 18S rRNA levels. PCR amplification to detect inducible NOS (iNOS) and the housekeeping 18S rRNA was performed with TaqMan gene expression assays (proprietary primers and probes designed and synthesized by Applied Biosystems). Data are presented as fold increase relative to measurements in vehicle-treated mice.

### Data Analysis

All data was analyzed using SigmaPlot 12.0 (Aspire Software International, Ashburn, VA). Data are presented as the mean ± SEM. Differences between groups were determined by ANOVA. The Holm-Sidak post hoc test was used to identify differences between specific groups. Paired comparisons were performed with the two-tailed Student's *t* test. The significance level was set at P<0.05.

## Results

### LPS Increases Plasma Cytokine Levels

**Figure 2:**
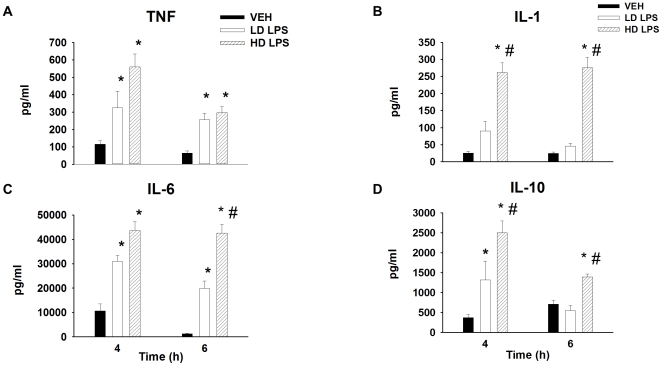
Plasma cytokine levels following vehicle and LPS treatment. Plasma concentration of tumor necrosis factor-alpha (TNF; Panel A), interleukin-1β (IL-1; Panel B), interleukin-6 (IL-6; Panel C) and interleukin-10 (Panel D; IL-10) 4- and 6-hours following vehicle (VEH), low-dose LPS (LD LPS; 1 µg/gBW), and high-dose LPS (HD LPS; 10 µg/gBW) treatment in chronically catheterized conscious C57/Bl6J mice, Data are expressed as mean±SEM. * p<0.05 vs. VEH, # p<0.05 vs. LD LPS.

Arterial plasma TNF-α, IL-6, IL-1β, and IL-10 levels were measured at two time points (4-, and 6-h post-vehicle or LPS injection), which represent the time points at the beginning and end of the hyperinsulinemic-euglycemic clamp. Cytokine levels were equal in the three groups during the basal period (data not shown). At 4 h after LPS injection, the mice that received low-dose LPS had significantly higher TNF- α (3-fold), IL-6 (3-fold), IL-1β (3-fold), and IL-10 (5-fold) above mice that received vehicle (Figure 1). The mice that received high-dose LPS also had significantly higher levels of TNF-α (7-fold), IL-6 (4-fold), IL-1β (7-fold), and IL-10 (6-fold) over vehicle-treated animals. Only IL-1β levels was significantly increased in high-dose LPS compared to mice that received low-dose LPS (2-fold). At 6 h post-LPS injection only TNF-α and IL-6 remained significantly increased in mice having received low dose LPS compared to vehicle-treated mice (3- and 6-fold respectively). In mice that received high-dose LPS significant increases in TNF-α, IL-6, IL-1β, and IL-10 still remained 6 h post injection compared to vehicle-treated animals (4-, 12-, 8-, and 2-fold respectively) and in IL-6, IL-1β and IL-10 compared to low-dose LPS treated animals (2-, 4- and 2-fold respectively; Figure 2).

### LPS Inhibits Insulin-Stimulated Muscle Glucose Uptake

**Figure 3:**
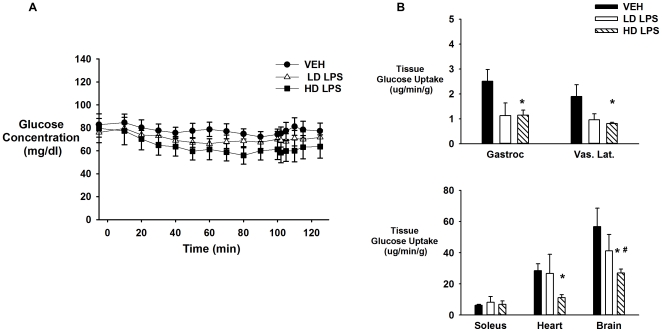
Basal glucose concentrations and tissue glucose uptake following vehicle and LPS treatment. Basal arterial glucose concentrations (Panel A) and tissue glucose uptake (Panel B) in chronically catheterized 5-hour fasted C57/BL6j mice that received vehicle (VEH), low-dose LPS (LD LPS; 1 µg/gBW), or high-dose LPS (HD LPS; 10 µg/gBW). Data are expressed as mean±SEM. * p<0.05 vs. VEH, # p<0.05 vs. LD LPS.

There was no significant difference in the body weight (grams) between mice that received vehicle, low-, or high-dose LPS (25.3±0.8, 26.6±0.8, and 25.3±0.7, respectively). During the saline infusion, there was no significant difference in fasting arterial glucose concentrations between mice that received vehicle, low-, or high-dose LPS (Figure 3A). However, insulin levels (ng/mL) were significantly lower 4-h post injection (t = 0 min; 0.51±0.10 vs. 0.17±0.01) and 6-h post injection (t = 125 min; 0.47±0.05 vs. 0.22±0.04) with high-dose LPS administration, while there was an increase with low-dose LPS administration at t = 0 min (0.51±0.10 vs. 0.88±0.07) and t = 125 min (0.47±0.05 vs. 1.16±0.22). Basal endogenous glucose production (mg·kg^−1^·min^−1^) was decreased in mice that received both low- and high-dose LPS compared to vehicle treated animals (10±1.9, 7.4±1.2, and 6.3±0.9; vehicle, low-dose, and high-dose LPS, respectively). Basal tissue glucose uptake was also assessed in these mice. Basal tissue glucose uptake was significantly decreased by high-dose LPS in all tissues in which it was assessed except for the soleus. The response in the low-dose group was highly variable which prevented us from detecting a decrease in basal tissue glucose uptake (Figure 3B).

**Figure 4:**
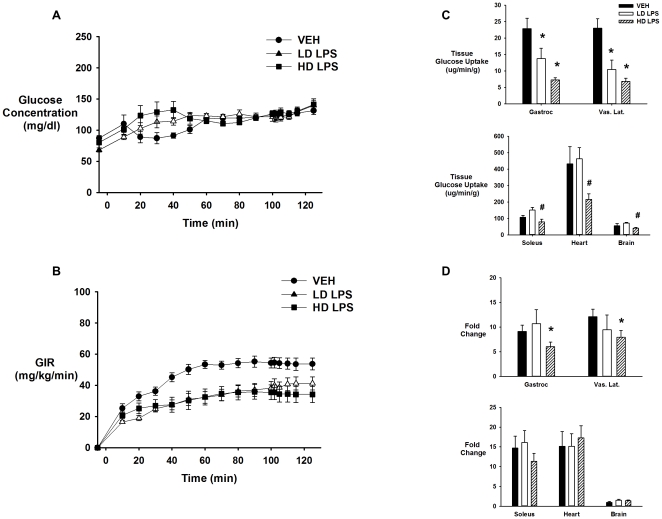
Glucose concentrations and insulin-stimulated tissue glucose uptake following vehicle and LPS treatment. Arterial glucose concentrations (Panel A); glucose infusion rate (GIR; Panel B) and tissue (soleus, gastrocnemius, superficial vastus lateralis (SVL), heart, and brain) glucose uptake (Panel C), and fold-change from basal of insulin-stimulated glucose uptake (Panel D) during a hyperinsulinemic-euglycemic clamp in chronically catheterized 5-hour fasted C57/BL6j mice that received vehicle (VEH), low-dose LPS (LD LPS; 1 µg/gBW), or high-dose LPS (HD LPS; 10 µg/gBW),. Data are expressed as mean±SEM. * p<0.05 vs. VEH, # p<0.05 vs. LD LPS.

Insulin action was assessed in mice that received vehicle, low-dose, or high-dose LPS under hyperinsulinemic-euglycemic clamp conditions (Table 1). Because LPS decreases insulin clearance, the insulin infusion rates were varied between the three groups to match insulin concentrations. While glucose levels were maintained at ∼120 mg/dl between all groups, there was a significant difference in the glucose infusion rate (GIR; mg•kg^−1^•min^−1^) necessary to maintain euglycemia between the mice that received vehicle and mice that received low- and high-dose LPS (54.2±0.5 vs. 38.6±1.1 vs. 35.4±0.6; Figure 4A and 4B). Endogenous glucose production was below zero in all groups indicating complete suppression of liver glucose production by insulin. Low-dose LPS markedly inhibited Rg in the gastrocnemius and SVL muscles with no significant difference in any other tissues measured compared to vehicle. High-dose LPS inhibited Rg in all tissues measured (Figure 4C). However, when Rg was compared to the fold-change over basal, low-dose LPS did not lead to a significant decrease in the fold increase in glucose uptake in any of the tissues measured, while mice that received high-dose LPS, still had a significant decrease in the fold increase in glucose uptake in the SVL and gastrocnemius muscles when compared to vehicle (Figure 4D).

**Table 1:** Arterial plasma insulin levels after a hyperinsulinemic-euglycemic clamp following vehicle or LPS treatment.

Time (min)	0	125
**Insulin (ng/ml)**		
Vehicle	0.33±0.09	2.54±0.19
Low-Dose LPS	0.88±0.07	3.04±0.30
High-Dose LPS	0.32±0.04	3.73±0.25

Arterial plasma insulin levels in response to vehicle, low- (1 µg/gBW), or high- (10 µg/gBW) dose LPS in chronically catheterized conscious mice during the basal period (0 min) and 125 min after initiation of a hyperinsulinemic-euglycemic clamp. Data are expressed as mean±SEM.

### LPS Does Not Decrease Insulin Signaling

**Figure 5:**
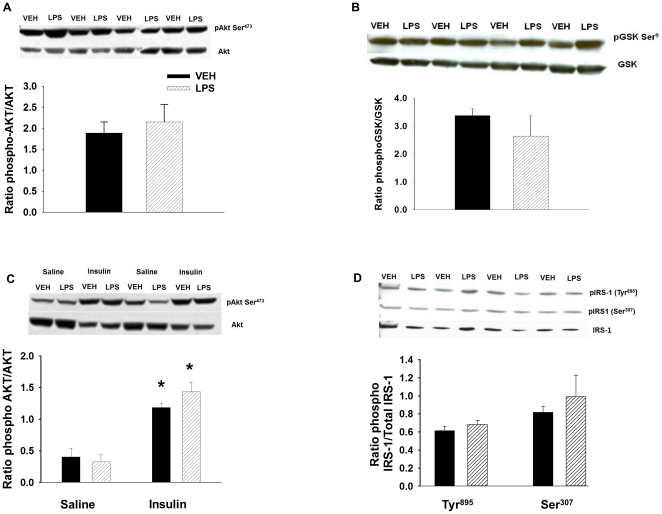
Effect of vehicle and LPS treatment on insulin signaling. Akt phosphorylation (Ser^473^; Panel A) and GSK phosphorylation (Panel B) in the gastrocnemius muscle of C57/BL6j mice that received vehicle (VEH) or high-dose LPS (HD LPS; 10 µg/gBW) after a hyperinsulinemic-euglycemic clamp. Akt phosphorylation (Ser^473^; Panel C) and phosphorylation of IRS-1 (Tyr^895^ and Ser^307^; Panel D) in the gastrocnemius muscle of C57/BL6j mice after an acute bolus of saline or insulin. Data are expressed as mean±SEM. *p<0.05.

We assessed the effects of high-dose LPS treatment on insulin signaling through the PI3 kinase pathway in the gastrocnemius muscle by measuring insulin-mediated phosphorylation of Akt, a key protein involved in insulin-stimulated MGU. When we compared levels of pAkt (Ser^473^) between control and LPS-treated mice from tissues obtained at the end of the clamp, we found there was no significant difference between the two groups (Figure 5A). Similar results were seen for phosphorylation of the downstream protein GSK (Figure 5B).To determine if LPS altered the phosphorylation of substrates in the PI3 kinase pathway acutely, 4 h after LPS or vehicle treatment mice received a bolus of insulin (10 U/kg body weight) and were sacrificed 10 min later. There was no significant difference in the acute phosphorylation of IRS-1 or Akt signaling between the vehicle- and LPS-treated mice (Figure 5C and 5D).

### LPS Increases iNOS Expression in Muscle

**Figure 6:**
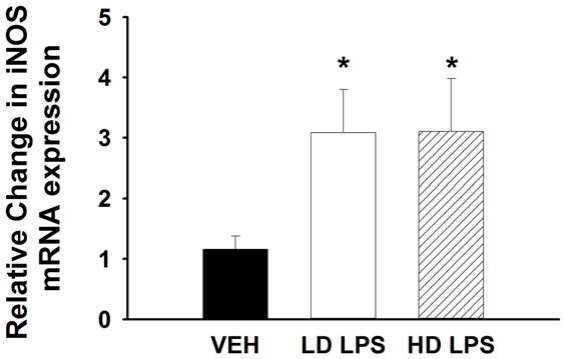
Effect of vehicle and LPS treatment on the expression of iNOS. Expression of inducible nitric oxide synthase (iNOS) mRNA in the gastrocnemius muscle of C57/BL6j mice that received vehicle, low-dose LPS (1 µg/gBW), or high-dose LPS (10 µg/gBW) after a hyperinsulinemic-euglycemic clamp. Data are expressed as mean±SEM. *p<0.05.

We examined the effect of LPS administration on the expression of inducible nitric oxide synthase (iNOS) in the skeletal muscle from vehicle- and LPS-treated mice. LPS significantly increased the expression of iNOS mRNA to a similar extent in the muscle in both low- and high-dose LPS treated mice compared to vehicle controls (Figure 6).

### High-Dose LPS Treatment Decreases Muscle Blood Flow

Mean arterial blood pressure (mmHg) was unchanged in vehicle treated mice. High-dose, but not low-dose, LPS lead to a significant reduction in mean arterial blood pressure (115.8±5.3 vs. 96.6±5.3 [vehicle]; 119.2±3.9 vs. 86.5±3.9 [low-dose LPS], 123.7±3.2 vs. 77.4±3.2 [high-dose LPS], −240 min vs. 0 min, respectively). We next determined if high-dose LPS treatment caused a similar reduction in blood flow. High-dose LPS treatment led to a ∼65% decrease in renal blood flow compared to vehicle treated mice. This also resulted in ∼65% decrease in muscle blood flow (Table 2). There was no significant difference in blood flow between the right and left kidney.

**Table 2:** Renal and tissue blood flow following vehicle or LPS treatment.

	VEH	LPS
**Renal blood flow (ml·kg BW^−1^ min^−1^)**		
	363±94	126±8*
**Tissue blood flow (ul·g tissue^−1^·min^−1^)**		
Soleus	6805±3440	2219±659*
Gastrocnemius	583±134	194±29*
SVL	976±183	359±88*
Heart	1389±362	416±53*

Renal and muscle (soleus, gastrocnemius, superficial vastus lateralis, and heart) blood flow were compared in mice that received either vehicle (VEH) or high-(10 µg/gBW) dose LPS. Data are expressed as mean±SEM.

*p<0.05.

### High-Dose LPS Impairs Cardiac Function

The effect of LPS-treatment on cardiovascular parameters and whole body oxygen consumption (VO_2_) was examined. There were no significant differences in stroke volume, heart rate, and cardiac output in vehicle and low-dose LPS treated mice 6 h after treatment. VO_2_ was not decreased in the low-dose LPS treated mice compared to vehicle. However, the administration of high-dose LPS led to a ∼30% decrease in heart rate, ∼30% decrease in stroke volume, a ∼50% decrease in cardiac output, and a ∼50% decrease in VO_2_ (Table 3).

**Table 3:** Cardiovascular parameters following vehicle or LPS treatment.

Time (h)	0	6
**Stroke Volume (uL)**		
Vehicle	36±3	48±7
Low Dose LPS	63±9	51±6
High Dose LPS	48±2	32±2†
**Heart Rate (bpm)**		
Vehicle	720±14	648±21†
Low Dose LPS	667±4	667±28
High Dose LPS	678±19	450±49† * #
**Cardiac Output (ml·g^−1^·min^−1^)**		
Vehicle	0.67±0.07	0.82±0.16
Low Dose LPS	1.21±0.19	0.99±0.12
High Dose LPS	0.91±0.17	0.42±0.12† * #
**VO_2_ (ml·kg^−1^·h^−1^)**		
Vehicle	3187±161	3044±249
Low Dose LPS	3346±116	2636±293†
High Dose LPS	3212±61	1653±218† * #

Comparison of stroke volume, heart rate, cardiac output, and oxygen consumption (VO_2_) in mice at baseline (t = 0) and 6 h after receiving vehicle, low- (1 µg/gBW), or high- (10 µg/gBW) dose LPS. Data are expressed as mean±SEM.

† p<0.05 vs. baseline;

*p<0.05 vs. VEH at 6 h;

# p<0.05 vs. low-dose LPS at 6 h.

## Discussion

Skeletal muscle represents an important site for insulin-stimulated glucose disposal. It is well known that insulin-stimulated muscle glucose uptake is impaired in the presence of inflammatory mediators. While it has been suggested from *in vitro* studies that the underlying factors leading to this impairment is defective insulin signaling, the mechanism *in vivo* remains to be clearly elucidated [16], [17]. The aim of the present study was to use a conscious mouse model to test the hypothesis that there was a significant vascular component to impaired insulin action *in vivo* resulting from a LPS challenge. Our findings indicate that the inflammatory stress induced by LPS in the mouse impairs MGU *in vivo* without affecting insulin signaling. The impairment in MGU was associated with decreases in muscle blood flow, suggesting that alterations in muscle glucose delivery play a critical role in insulin resistance when the cardiovascular system is altered.

Hyperinsulinemic-euglycemic clamps in chronically catheterized conscious mice were used to determine insulin-stimulated glucose uptake. In initial studies vehicle and high-dose LPS treated mice were both infused with 4 mU·kg^−1^·min^−1^ of insulin during the clamp. There was no difference in glucose requirements between the groups. However, arterial insulin was two-fold higher in mice that received LPS (5.0 vs. 2.5 ng/ml; LPS vs. vehicle) implying that LPS impaired insulin clearance. In subsequent studies the insulin infusion rate was reduced to 2.5 mU·kg^−1^·min^−1^ in an attempt to match circulating insulin levels in LPS treated mice to those having received vehicle. Although insulin levels remained somewhat higher, glucose requirements were reduced by 35% and there was a significant decrease in glucose uptake in multiple muscles. When the dose of LPS was decreased to 1 mg/kg impairments in insulin-stimulated MGU were not as robust. These results are consistent with findings in a variety of species [18], [19], [20]. Our results suggest that decreased insulin clearance by LPS may serve to normalize insulin-stimulated glucose uptake. A recent study Park *et al.* found that during a hyperinsulinemic-euglycemic clamp LPS did not affect insulin action *in vivo* [10]. In the presence of LPS, there was either an increase or no difference in the glucose infusion rate versus animals that received saline. Similar results were seen when they examined whole-body glucose disposal. They found that while LPS lead to a decrease in hepatic glucose production, muscle glucose uptake was unaltered. Interestingly insulin concentrations were not reported [10]. Pilon *et al.* did observe a decrease in whole body glucose uptake during the hyperinsulinemic-euglycemic clamp 6 h after administering LPS. In contrast to our study, insulin-stimulated glucose uptake by the soleus (other tissues were not evaluated) was markedly decreased however they utilized a higher dose of LPS than our high dose group (20 mg/kg). Like Park *et al.*, they also did not report insulin concentrations during the clamp [10], [16]. We found that LPS impaired insulin clearance therefore the insulin infusion rate during the clamp was decreased to maintain similar arterial insulin concentrations between groups. Thus, it is likely that in their study impairments in insulin clearance and the consequent increase in arterial insulin concentrations following LPS treatment offset any underlying impairment in insulin stimulated tissue glucose uptake.

Defects in insulin signaling through the PI3-kinase pathway in the muscle during a LPS challenge do not contribute to impaired glucose uptake. Consistent with our previous work LPS treatment increases the plasma levels of inflammatory cytokines [21]. de Alvaro *et al.* found that in cultured rat skeletal muscle cells TNF-α lead to a decrease in insulin signaling through the IR/IRS-1/AKT pathway [22]. Impairments in insulin signaling are mediated in part by increased cytokines and subsequent NFκB pathway activation that occurs in response to LPS [23], [24]. We observed an increased expression of iNOS in muscle, which is consistent with recent reports [16]. Despite the robust activation of the inflammatory response, Akt phosphorylation in gastrocnemius muscle at the end of the clamp was unaltered by LPS. Pilon *et al.* observed a decrease in insulin stimulated IRS-1 associated PI 3-kinase activity in soleus (other muscles were not examined) obtained from control and LPS treated mice (6 h after LPS administration) stimulated *ex vivo* with insulin [16]. *Ex vivo* the vascular-dependent effects of insulin are absent. While *in vitro* models can unmask insulin signaling defects, it prevents detection of any vascular dependent alterations in insulin action. An additional consideration is that the impairment in insulin signaling may be dependent upon the time of exposure to the inflammatory environment, the dose of LPS administered, and the specific muscle group examined. Pilon examined insulin action utilizing a 2-fold higher dose of LPS than our high-dose group [16]. To assess whether the peak phosphorylation events were missed during the 120 min clamp study, Akt phosphorylation was examined 10 minutes following a bolus of insulin. This experiment confirmed the findings from the insulin clamp studies; LPS administration *in vivo* did not blunt insulin signaling in the muscle. Since Akt phosphorylation can be influenced by multiple events, we next looked upstream in the insulin signaling pathway at IRS-1 phosphorylation. We found that LPS administration did not blunt tyrosine phosphorylation nor did it increase serine phosphorylation of IRS-1 compared to vehicle treatment. These data suggest that there is dissociation between insulin-stimulated muscle glucose uptake and phosphorylation of key proteins in the PI3 kinase insulin signaling pathway. This strongly suggests that other factors can limit muscle glucose uptake *in vivo*.

Consistent with the insulin signaling data we found that despite the absolute decrease in MGU the fold increase above basal MGU was unaltered in several tissues. Insulin-stimulated MGU increased ∼10 fold above vehicle infused mice irrespective of whether they are given LPS. This suggests that the tissues were very responsive to insulin but that other factors such as a decrease in metabolic demand or vascular delivery of glucose limited the absolute magnitude of glucose uptake. Cardiac output following low-dose LPS was not significantly altered. However, we found that high-dose LPS significantly decreased cardiac output by ∼50% and mean arterial blood pressure fell by ∼50%. In conjunction with these results, we found that VO_2_ was significantly decreased with high-dose LPS (∼50%) while low-dose LPS (∼20%) did not significantly decrease metabolic demand (i.e. VO_2_). A correlation between metabolic demand and insulin stimulated glucose uptake is well established as increasing metabolic demand can compensate for underlying insulin resistance [25], [26], [27]. This may in fact explain why in humans it has been reported that during endotoxemia insulin can initially improve insulin action [28]. The low dose of LPS used in human studies induced modest cardiovascular alterations (tachycardia and mild hypotension). In contrast to the mouse in both the human and pig the dose of LPS used caused hyperthermia, presumably increasing the metabolic rate and limiting cardiovascular disturbances. Therefore depending on the species, the duration of exposure to, and the dose of LPS used, among other factors such as the metabolic rate and associated cardiovascular events, may offset any negative effects of LPS on tissue glucose uptake. Thus, the combined decrease in tissue blood flow and VO_2_ following LPS may explain the accompanying impairment in MGU seen in our mouse model. Low dose LPS had limited alterations in cardiac output yet they were insulin resistant. This could be due to subtle changes in tissue perfusion that combine with other defects downstream of AKT signaling (e.g. glucose transport or phosphorylation). Our data suggest that the accompanying cardiovascular events should be considered when interpreting the impact of LPS *in vivo*.

Previous studies have shown that increased blood flow leads to increased glucose uptake provided the muscle membrane is sufficiently permeable to glucose, such as it is during insulin stimulation [29], [30]. The impairment in MGU following LPS administration could be due to the combined effects of reduced tissue blood flow and/or extraction of glucose. Using PAH and microspheres to measure tissue blood flow we found that after high-dose LPS muscle blood flow was significantly impaired compared to vehicle-treated animals. This would decrease muscle glucose delivery and in the absence of a compensatory increase in fractional glucose extraction, MGU. Blood flow and glucose uptake decreased proportionally in both the SVL and gastrocnemius muscle (∼69%), which suggests that fractional glucose extraction was unaltered. In contrast, blood flow to the heart and soleus decreased to a greater extent (∼70% and 65%, respectively) than the fall in glucose uptake (∼50% and 25%, respectively). This suggests that these tissues had a compensatory increase in fractional extraction of glucose and were able to minimize the decrease in tissue glucose uptake. As fat is the preferred substrate in these tissues they may switch to glucose utilization as the preferred substrate in low flow states. Indeed, studies by Petersen *et al.* demonstrated that the extracellular barrier to glucose uptake was lower in Type I muscle fibers [31]. Thus tissues with greater oxidative capacity and capillary densities (heart and soleus) have a greater ability to extract glucose in setting of insulin resistance and low flow states.

An inflammatory state can lead to decreased microcirculatory flow velocity and the density of perfused capillaries in rat models both of which are important factors in determining MGU [32], [33]. Our data suggests that high-dose LPS decreases blood flow to the muscle which subsequently impairs MGU. This is consistent with data that there is a redistribution of blood flow during inflammatory stress [34]. Unlike glucose, decreased muscle insulin delivery would not necessarily result in decreased insulin signaling because insulin is not consumed by the muscle. Therefore the concentration of insulin presented to muscle cells would be unaltered in a setting of decreased tissue blood flow. The lack of difference in IRS and Akt phosphorylation between vehicle- and LPS-treated mice indicates a disassociation of insulin-stimulated glucose uptake and insulin signaling though the PI3 kinase pathway in muscle. Similar results in insulin signaling were observed by Orellana *et al.* in the neonatal pig [35]. The impairment in tissue blood flow following LPS administration is consistent with studies suggesting that skeletal muscle perfusion could act as an independent determinant of insulin stimulated glucose uptake [3], [29], [36]. The brain is not an insulin responsive tissue therefore during a hyperinsulinemic-euglycemic clamp we would not expect a decrease in the glucose uptake. Therefore a fall in brain glucose uptake under these conditions is also consistent with a decrease in tissue blood flow. Pilon *et al.* observed that mice lacking iNOS are protected from LPS-induced insulin resistance due to diminished nitrosylation of IRS-1 in the soleus muscle [16]. However, an equally plausible explanation is that these mice are protected from the hypotension and the decrease in tissue perfusion that accompanies the excess nitric oxide generation from LPS [37], [38].

In summary, this study provides evidence that an LPS challenge that impairs cardiovascular function significantly impairs MGU in mice but does not affect the stimulation of insulin signaling *in vivo* despite the release of inflammatory cytokines. Thus impairments in effective tissue perfusion can contribute to the decreased glucose uptake observed during endotoxemia.
